# Spatio-temporal tumor heterogeneity in metastatic CRC tumors: a mutational-based approach

**DOI:** 10.18632/oncotarget.26081

**Published:** 2018-09-28

**Authors:** Sofía del Carmen, José María Sayagués, Oscar Bengoechea, María Fernanda Anduaga, Jose Antonio Alcazar, Ruth Gervas, Jacinto García, Alberto Orfao, Luis Muñoz Bellvis, María Eugenia Sarasquete, María del Mar Abad

**Affiliations:** ^1^ Department of Pathology and IBSAL, University Hospital of Salamanca, Salamanca, Spain; ^2^ Cytometry Service-NUCLEUS, Department of Medicine, Cancer Research Center (IBMCC-CSIC/USAL) and IBSAL, University Hospital of Salamanca, Salamanca, Spain; ^3^ General and Gastrointestinal Surgery Service and IBSAL, University Hospital of Salamanca, Salamanca, Spain; ^4^ Hematology Service, University Hospital of Salamanca, Salamanca, Spain

**Keywords:** colorectal cancer, clonal evolution, anti-EGFR, mutational profile, tumor heterogeneity

## Abstract

It is well known that activating mutations in the *KRAS* and *NRAS* genes are associated with poor response to anti-EGFR therapies in patients with metastatic colorectal cancer (mCRC). Approximately half of the patients with wild-type (WT) *KRAS* colorectal carcinoma do not respond to these therapies. This could be because the treatment decision is determined by the mutational profile of the primary tumor, regardless of the presence of small tumor subclones harboring RAS mutations in lymph nodes or liver metastases. We analyzed the mutational profile of the *KRAS*, *NRAS*, *BRAF* and *PI3KCA* genes using low-density microarray technology in samples of 26 paired primary tumors, 16 lymph nodes and 34 liver metastases from 26 untreated mCRC patients (n=76 samples). The most frequent mutations found in primary tumors were *KRAS* (15%) and *PI3KCA* (15%), followed by *NRAS* (8%) and *BRAF* (4%). The distribution of the mutations in the 16 lymph node metastases analyzed was as follows: 4 (25%) in *KRAS* gene, 3 (19%) in *NRAS* gene and 1 mutation each in *PI3KCA* and *BRAF* genes (6%). As expected, the most prevalent mutation in liver metastasis was in the *KRAS* gene (35%), followed by *PI3KCA* (9%) and *BRAF* (6%). Of the 26 cases studied, 15 (58%) displayed an overall concordance in the mutation status detected in the lymph node metastases and liver metastases compared with primary tumor, suggesting no clonal evolution. In contrast, the mutation profiles differed in the primary tumor and lymph node/metastases samples of the remaining 11 patients (48%), suggesting a spatial and temporal clonal evolution. We confirm the presence of different mutational profiles among primary tumors, lymph node metastases and liver metastases. Our results suggest the need to perform mutational analysis in all available tumor samples of patients before deciding to commence anti-EGFR treatment.

## INTRODUCTION

Sporadic colorectal cancer (sCRC) is the third most frequently diagnosed cancer worldwide and the third most common cause of cancer-related death [1]. Approximately 300,000 new cases of sCRC are reported each year and 200,000 patients (67% of cases) die from cancer-related complications, most of them as a consequence of the metastatic process (i.e., mostly liver metastasis) [2]. In recent years, therapies with anti-EGFR agents targeting the metastatic process have improved sCRC outcome, but only a subset of selected patients benefit from these treatments [3]. It is well known that activating mutations in the *KRAS* (35% of cases) and *NRAS* (1-3%) genes are associated with poor response to anti-EGFR therapies, which rules out anti-EGFR-directed therapy as an option for these patients [4]. The *BRAF* V600E mutation occurs in 10-15% of metastatic CRC (mCRC) cases [5], but does not predict resistance to anti-EGFR therapies. However, *BRAF* mutation is a strong marker of poor prognosis in mCRC [5]. Genetic events in additional nodes of the EGFR pathway, such as *PI3KCA* exon 20 mutations, may also confer resistance to anti-EGFR therapies [6, 7]. In addition, up to 50% of the patients with wild-type (WT) *KRAS* colorectal carcinoma do not respond to this therapy, possibly because the decision to treat is determined by the mutational profile of the primary tumor, regardless of the presence of small tumor subclones harboring RAS mutations in lymph nodes or liver metastases. The most recent European Society for Medical Oncology (ESMO) guidelines for the management of patients with metastatic sCRC recommend that RAS mutation testing be performed in either primary tumor or liver metastasis samples, tissue from other metastatic sites, such as lymph node metastases, may be used if neither a primary tumor nor a liver metastasis sample are available [8]. Most studies have employed primary tumor samples from sCRC patients to define the mutations of *KRAS*, *NRAS*, *BRAF* and *PI3KCA* genes [9, 10]. However, apparently contradictory results have been reported about the concordance between the mutational status of these genes in primary tumors and their corresponding liver metastases. Thus, while several papers report full concordance between primary tumors and liver metastases [11–13], others have found up to 31% [14] of discrepancies. In addition, studies comparing the mutation distribution in primary tumors and lymph node metastases in the same patient are scarce [15, 16]. Our research group has recently provided detailed information, obtained from the use of FISH and high-density single-nucleotide polymorphism (SNP) array techniques, about the clonal heterogeneity and genetic diversity within a given tumor, whereby different abnormalities coexist in the same tumor sample (intratumoral heterogeneity) [17]. However, the precise patterns of intratumoral clonal evolution estimated from the mutational profile of the genes associated with the EGFR signaling pathway, and their relationship with the neoplastic clones present in primary *vs.* lymph node metastases *vs.* metastatic tumor samples (intertumoral heterogeneity) remain controversial. It is important to identify intratumoral and intertumoral heterogeneity since both could affect the response to targeted therapies.

Here, we analyzed the mutational profile of *KRAS*, *NRAS*, *BRAF* and *PI3KCA* genes using low-density microarray technology in samples of 26 paired primary tumors, 16 lymph nodes and 34 liver metastases from 26 untreated mCRC patients (n=76 samples). Our aims were: (1) to identify mutational profile differences between paired primary *vs.* lymph node metastases *vs.* multiple liver metastases samples that might explain resistance to therapies in WT primary tumor, and (2) to describe the spatial intratumoral heterogeneity and temporal clonal evolutionary processes.

## RESULTS

### Mutation frequencies and intratumoral and intertumoral genetic heterogeneity

In all DNA samples a successful amplification of the mutant or WT allele was obtained by PCR. Mutations in at least one of the analyzed genes and/or one tumor sample (primary tumor, lymph node metastases or liver metastases) were found in 13 patients (50% of cases). Overall, 37 mutations were detected in the 76 tumor samples (11 in primary tumors, 9 in lymph node metastases and 17 in liver metastases). The mutations found in primary tumors were most frequently of the *KRAS* and *PI3KCA* genes (each with 4/26 cases; 15%), followed by those of the *NRAS* (2/26; 8%) and *BRAF* (1/26; 4%) genes. The mutations in the 16 lymph node metastases were distributed as follows: 4 (25%) in *KRAS*, 3 (19%) in *NRAS* and 1 in each of the *PI3KCA* and *BRAF* genes (6%). As expected, the most prevalent mutation in liver metastases was in the *KRAS* gene (12/34; 35%), followed by the *PI3KCA* (3/34; 9%) and *BRAF* (2/34; 6%) genes.

Detailed information about each patient and their sample mutation profile is provided in Table 1. Of the 26 cases studied, 15 (58%) displayed an overall concordance in the mutation status detected in the lymph node metastases and liver metastases compared with primary tumor (13 cases were WT and the other two had a *KRAS* mutation: G12D and G12V), suggesting no clonal evolution. In contrast, in the other 11 patients (42%), the mutation profile differed between the primary tumor and lymph node/metastasis samples, suggesting intertumoral clonal evolution.

**Table 1:** Details of the clinical, biological and genetic characteristics and type of sample studied of each individual metastatic colorectal cancer patient analyzed in this study (n=26)

CLINICAL FEATURES										GENETIC PROFILE OF ANALYZED SAMPLES					
Patients	Gender	Age (Years)	CEA Serum levels (ng/ml)	Site of PT	Histological grade	PT size (cm)	TNM stage	Peritoneal metastasis	Other metastases	PT	Lymph node	LM #1	LM #2	LM #3	LM #4
1	M	60	29.4	Right colon	Well	3.8	T3N0M1	No	No	WT	-	WT	-	-	-
2	M	74	1484	Right colon	Well	4	T3N2M1	No	No	WT	WT	WT	-	-	-
3	M	76	44	Right colon	Moderate	5.5	T3N1M1	No	Lung	WT	WT	*KRAS* (G12D)	-	-	-
4	M	62	155.2	Right colon	Moderate	3	T3N2M1	No	Lung	*KRAS* (G12D)	*KRAS* (G12D)	*KRAS* (G12D)	-	-	-
5	M	69	7.6	Left colon	Well	3	T3N0M1	Yes	Lung	WT	-	WT	-	-	-
6	M	64	256	Left colon	Well	7	T3N0M1	No	No	WT	-	WT	-	-	-
7	M	61	2.3	Left colon	Well	5	T3N2M0	Yes	No	*KRAS* (G12D) *PI3K* (E545K/D)	*KRAS* (G12D)	*BRAF* (V600E)	*KRAS* (G12D) *PI3K* (H1047R)^*^	-	-
8	M	57	30.9	Left colon	Well	6	T3N1M1	No	No	*PI3K* (H1047R)	*BRAF* (V600E) *NRAS* (G12D)	*KRAS* (G12D)^*^	*PI3K* (H1047R)^*^	*KRAS* (G12D)^*^	.
9	F	67	233.7	Left colon	Well	7	T3N1M1	No	No	*KRAS* (G12D)	*KRAS* (G12D) *NRAS* (Q61K)	*KRAS* (G12D)	*KRAS* (G12D)	*KRAS* (G12D)	-
10	M	77	244.9	Left colon	Well	5.5	T3N1M1	No	No	WT	-	WT^*^	-	-	-
11	M	61	1.2	Left colon	Moderate	3	T2N0M0	No	No	WT	-	WT^*^	-	-	-
12	F	75	1145	Left colon	Moderate	9	T4N1M1	Yes	Lung	*NRAS* (G12D)	*NRAS* (Q61K)	*KRAS* (G12V)	-	-	-
13	F	58	501	Left colon	Moderate	5	T4N2M1	Yes	Lung	WT	WT	WT	-	-	-
14	M	72	45.4	Left colon	Poorly	4	T3N1M1	Yes	No	WT	WT	WT	-	-	-
15	F	48	32.9	Left colon	Poorly	4	T4N2M1	Yes	No	*PI3K* (E545K/D)	*KRAS* (G12D)	*BRAF* (V600E)	-	-	-
16	M	66	3.7	Rectum	Well	8.5	T3N0M0	No	No	WT	-	WT^*^	-	-	-
17	F	62	139	Rectum	Well	4	T3N0M1	No	No	WT	-	WT	-	-	-
18	M	74	6.4	Rectum	Well	5	T3N1M0	No	No	*BRAF* (V600E)	WT	*KRAS* (G12A)	-	-	-
19	M	75	589.2	Rectum	Well	4	T3N1M0	No	No	WT	*PI3K* (E545K/D)	WT^*^	-	-	-
20	M	77	58.3	Rectum	Well	9	T3N1M1	No	No	WT	WT	*PI3K* (E545K/D)	-	-	-
21	M	63	23.2	Rectum	Well	7	T3N2M1	No	No	WT	WT	WT	-	-	-
22	M	64	5.4	Rectum	Well	7	T4N0M0	No	No	WT	-	WT^*^	-	-	-
23	M	79	0.8	Rectum	Well	5	T4N0M1	No	No	*KRAS* (G12V)	-	*KRAS* (G12V)	-	-	-
24	F	76	149.8	Rectum	Moderate	2.5	T3N1M1	No	No	WT	WT	WT	WT	WT	WT
25	F	49	6.8	Rectum	Moderate	6.5	T3N1M1	No	No	*PI3K* (E545K/D)	WT	*KRAS* (G12V)	-	-	-
26	M	74	110	Rectum	Moderate	5	T4N0M1	No	No	*NRAS* (Q61H)	-	WT	-	-	-

Abbreviations: M: male; F: female; CEA: carcinoembryonic antigen; PT: primary tumor; LM: different liver metastases developed in each patient; well: well-differentiated adenocarcinoma; moderate: moderately differentiated adenocarcinoma; poor: poorly differentiated adenocarcinoma; WT: wild-type; -: not applicable. ^*^metachronous sample.

**Figure 1:**
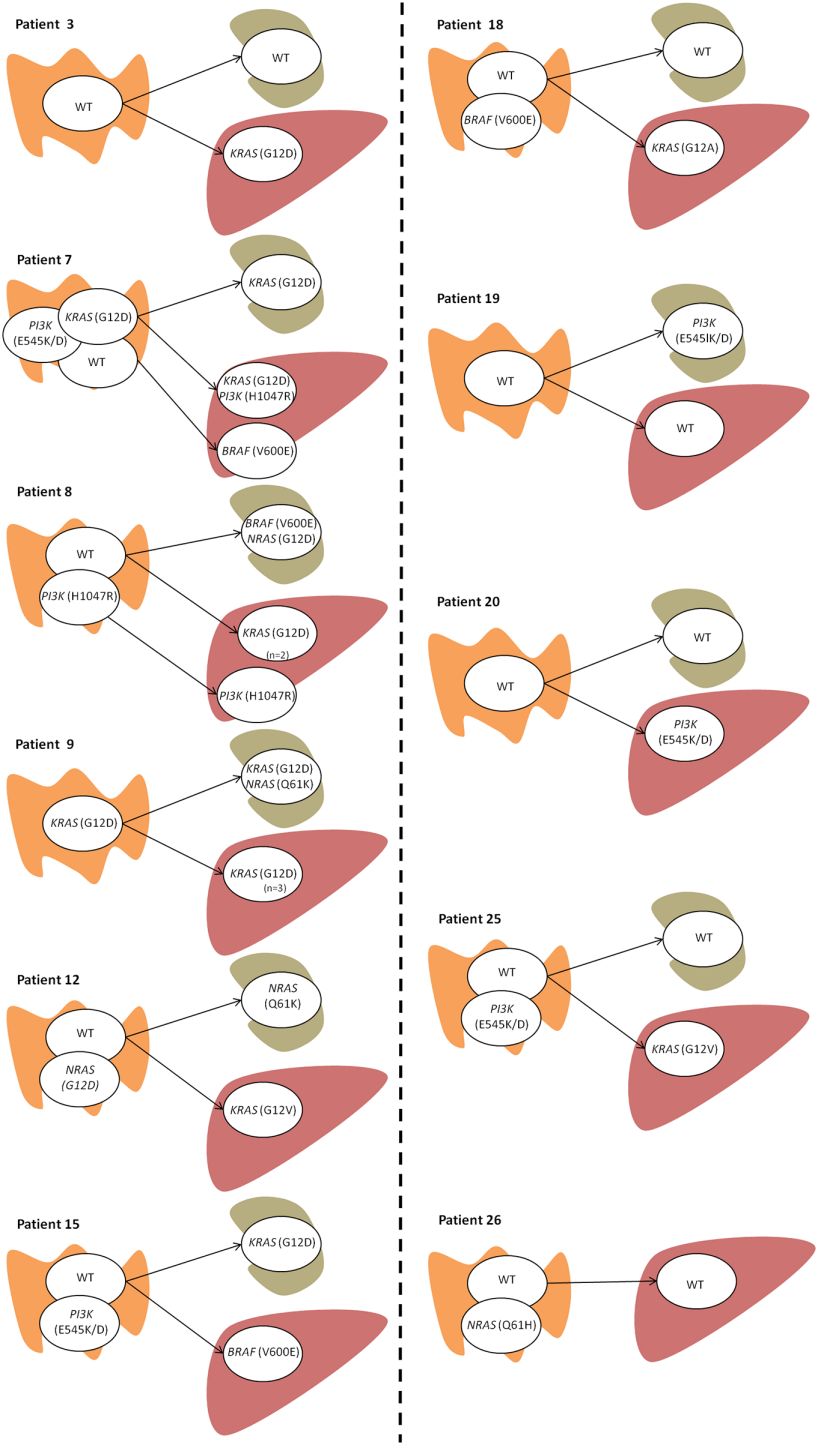
Intratumoral and intertumoral mutation heterogeneity of primary colorectal carcinomas as determined by the presence of different mutation status for *KRAS*, *NRAS*, *PIK3* and *BRAF* genes Only those patients with mutation heterogeneity across the three tumor samples in one or more of the four examined genes are shown (n=11). A detailed description of all patients (n=26) and samples (number and type) analyzed can be found in Table 1. Each circle represents a tumor cell clone detected in the sample analyzed. Tissue origin in represented by the following pictures: 
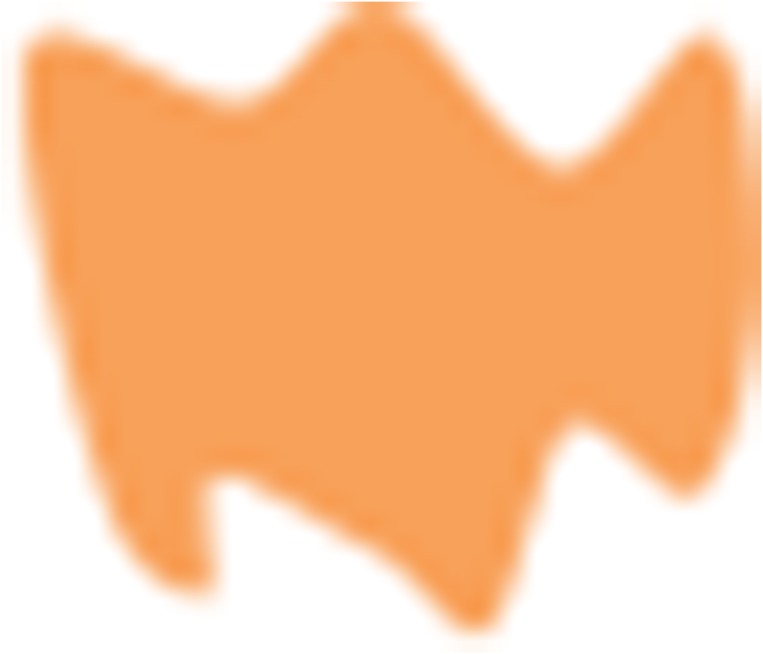
 for the primary tumor; 
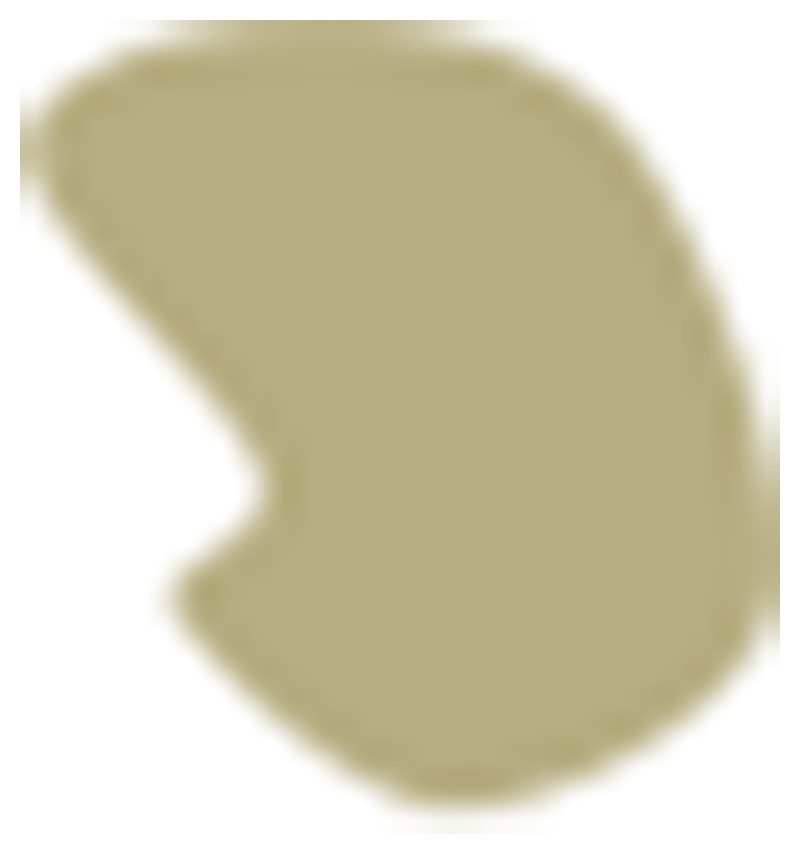
lymph node; 
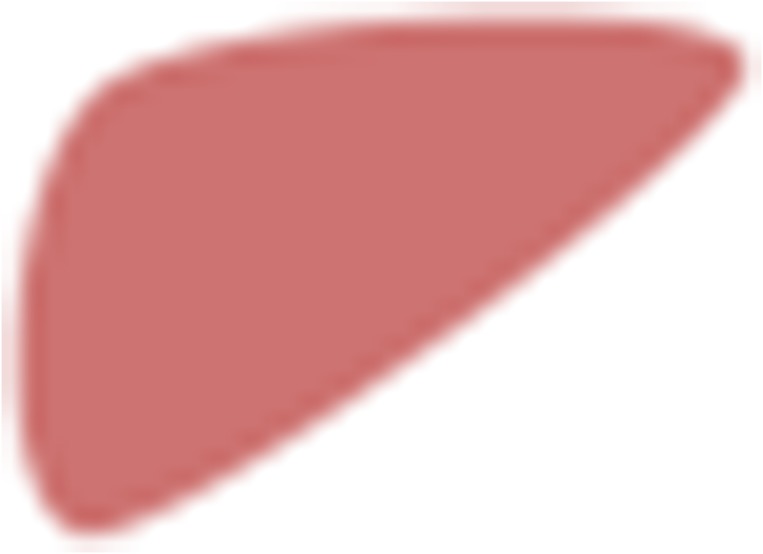
liver metastasis. If more than one sample per tissue was studied it is mentioned in the figure (n= number of samples).

Figure 1 summarizes the hypothetical pathways of intratumoral and intertumoral clonal evolution detected in these samples. The mutation profile identified in primary tumors was considered the founder clone portrait, the most common profile being characterized by the presence of two clones (n=6), one of which harbored a mutation in *PI3KCA* (n=3), *BRAF* (n=1) or *NRAS* (n=2), and the other one being a WT clone. However, there were four cases where the primary tumor was characterized by the presence of only one clone (n=4) —either a WT clone (n=3) or a mutated *KRAS* clone (n=1)— and another patient with three clonal populations in the primary tumor (a WT subclone and two subclones with *KRAS* and *PI3KCA* mutations). With respect to the lymph node metastasis status, all cases presented with one clonal population; four cases were WT and six cases harbored different mutations. Of the latter mutated cases, two were characterized by the presence of a *KRAS* mutation, and there were single cases each of an *NRAS* mutation, simultaneous *BRAF/NRAS* mutations, a *KRAS/NRAS* mutation, and a *PI3KCA* mutation. Only one of the six cases had a mutation concordant with its primary tumor sample.

Considering the liver metastases, nine cases showed one clonal population, the most frequent being a *KRAS*-mutated clone (n=5), followed by a WT clone (n=2), and *BRAF* (n=1) and *PI3KCA* (n=1) mutated clones. The two other cases had two clonal populations, the first with *KRAS* and *PI3KCA* mutated clones, and the second with one clone featuring a *KRAS* plus *PI3KCA* mutation and a second clone exhibiting a *BRAF* mutation.

### Clonal evolution patterns

Two patterns of clonal evolution are plausible from the evidence of these samples: (1) no evolution, when the mutation profile in the primary tumor is the same as in the lymph node metastases or liver metastasis sample and (2) linear evolution, when the primary tumor profile acquires additional mutations during the process of migration to the lymph node or liver.

Regarding the transition from primary tumor to the lymph node metastases, five cases showed no clonal evolution (patients 3, 7, 18, 20 and 25) and five cases displayed linear evolution (patients 8, 9, 12, 15 and 19). For the cases of primary tumor to liver metastasis, no evolution pattern was observed in three (patients 9, 19 and 26), and linear evolution was detected in seven of them (patients 3, 7, 12, 15, 18, 20 and 25). One case showed a mixed pattern (patient 8), whereby a WT clone acquired a *KRAS* mutation that was present in two liver metastasis samples (linear evolution) and another *PI3KCA*-mutated clone without any evolution.

## DISCUSSION

Approximately 30% of patients with sCRC have *KRAS* mutations and are resistant to EGFR inhibitors, and up to 50% of metastatic colorectal cancer (mCRC) cases with *KRAS* wild-type (WT) do not respond to anti-EGFR therapies [18, 19]. The presence of a *KRAS* WT genotype in the primary tumors from mCRC patients does not guarantee any benefit from EGFR inhibitors [19], so the availability of other factors that could predict treatment resistance is of great importance for identifying which *KRAS* WT mCRC patients will be non-responders and who will develop resistance after an initial response.

The aim of this study was to describe the intertumoral heterogeneity of mCRC tumors through the mutation frequency of four key driver genes (*KRAS*, *NRAS*, *PIK3CA* and *BRAF*) in 76 samples from 26 untreated mCRC patients at diagnosis corresponding to 26 primary tumors, 16 lymph node metastases and 34 liver metastasis samples and then to establish the intratumoral pathways of clonal evolution. To the best of our knowledge, this is the first study to propose and test a hypothetical model of intratumoral and intertumoral clonal evolution of the most frequently altered genes (*KRAS*, *NRAS*, *PIK3CA* and *BRAF*), comparing the mutational status in liver metastases, lymph node metastases versus paired primary colorectal tumors, using low-resolution arrays. Overall, our results show that colorectal cancers are highly heterogeneous tumors at the intratumoral and intertumoral genetic levels, which probably affects the response to targeted anti-EGFR agents.

Primary tumor heterogeneity is a well-recognized challenge to personalized medicine [20–22]. Previous studies have revealed clonal heterogeneity of *KRAS* mutations within primary mCRC [23, 24] and regarding the concordance in mutation status between primary tumors and metastatic deposits [25, 26]. However, mutational heterogeneity across synchronous deposits and lymph node metastases is not well described. Taking *KRAS* mutations as an example, some studies showed 100% concordance between primary CRC tumors and paired metastases [12, 13], while others reported 4-30% discordance [14, 15]. Losi *et al.* [11] studied primary tumors and paired metastases from 35 patients, and found that *KRAS* mutations were present in 71% of cases with 100% concordance, even in the local recurrences of the same patient. However, the literature suggests an incidence of *KRAS* mutations in approximately 45% of cases with mCRC [9, 27, 28]. In addition, the series analyzed by Knijn et al. (n=305 patients) showed 96% concordance in the *KRAS* gene status between primary tumor and paired liver metastases [29]. This finding is consistent with KRAS mutations mostly occurring as an early molecular event. On the other hand, there is increasing evidence of a degree of discordance in the *KRAS* mutational status between different tumoral samples from the same patient. Recently, Jeantet *et al*. [30] reported that 33% of cases with spatial intratumoral heterogeneity for *RAS* mutations coexisted within the same tumor with *KRAS* and/or *NRAS* mutated and WT zones. Kosmidou *et al.* [31] found similar intratumoral heterogeneity of *KRAS* mutations (44% discordance) when they compared tumor center and tumor periphery. Al-Mulla *et al.* [16] analyzed *KRAS* status in 26 liver metastases and 31 lymph node samples and detected an overall discordance rate of 19% in the two subgroups compared with the primary tumor. Furthermore, the discordance reached 30% in the series analyzed by Albanese *et al.* [14]. Likewise, Miranda *et al.* [32] found different rates of *KRAS* mutations in lymph nodes (19%), liver metastases (38%) and primary tumors (32%) in 101 sCRC patients. The lower mutation rate in lymph nodes compared with primary tumors may indicate that neoplastic cells colonizing lymph nodes leave the primary tumor before *KRAS* alterations occur, or, if it is assumed that the primary tumor comprises different cell clones, one of these clones is responsible for survival and proliferation in the cells of the primary site. On the other hand, the presence of higher rates of *KRAS* mutations in liver metastases can be explained by the gradual acquisition of mutations as a secondary alteration during disease progression.

Other genes involved in the EGFR signaling pathway (*NRAS, BRAF* and *PIK3CA*) have been widely studied in primary sCRC. Mao *et al*. in their review and meta-analysis [26], examined the concordance of *KRAS*, *BRAF*, and *PIK3CA* mutational status between primary tumors and metastases, finding high concordance rates among liver metastases with primary tumor, but low concordance for the three biomarkers with lymph node metastases. Baldus *et al*. [15] studied 100 patients and found major discordances in primary tumor versus lymph node metastases, whereby 17 out of 55 patients (31%) whose *KRAS* mutation profile was examined had a discordant result. This heterogeneity was also found in 4% and 13% of cases for *BRAF* and *PIK3CA*, respectively. As we have previously shown with FISH and high-density single-nucleotide polymorphism (SNP) array techniques [17], the metastases from an individual with mCRC are extremely similar to each other but are divergent from the paired primary tumor. Particular clonal genetic events present in the metastasis samples can also be found in restricted subclones of the primary tumor, suggesting that only some tumor cells within the primary tumor have the ability to metastasize, as is sometimes observed in human medulloblastoma [33]. Another explanation for our findings is that the primary tumor may have been reseeded by a metastatic clone that had experienced additional genetic events at the periphery. In our series, mutations of the *KRAS* gene were more frequent in liver metastases (36%) than in primary tumors (16%). The greater incidence of *KRAS* mutations in liver metastases implies that they are acquired mutations. There is a general consensus that cancer progression arises from a single mutated cell, followed by a clonal expansion associated with genetic alterations. The acquisition of these alterations can result in the emergence of new tumor subclones with different genotypes. In this context, we observed that 13 out of 16 WT primary tumors did not change their molecular profile during disease progression, which means that it is more likely that WT primary tumors will not clonally evolve. This may be because they are made up of a single non-mutated clone and are genetically stable; several studies have examined prognostic markers of outcome after liver resection for colorectal cancer metastases and concluded that genetic stability is associated with a better outcome [34]. In addition, we observed that in 5 of the 11 cases showing linear clonal evolution between the primary tumor and the lymph node metastasis, four were WT clones. As previously discussed [15, 32], the lower incidence of mutations in lymph node metastases indicates that these metastases are caused by tumor clones that escape from the primary tumor in the early stages of the disease, before *KRAS* or other mutations occur. In addition, clonal selection during the metastatic process is noted, since the mutations in *PIK3CA* are not observed during tumor progression (only two cases had acquired *PIK3CA* mutations in liver metastases). Clones with mutated *KRAS* are observed from the outset or during the metastatic process. It is well known that *RAS* and *BRAF* mutations are mutually exclusive [35], but our study shows that the presence of either a *KRAS* or a *BRAF* mutation in primary tumor or at metastatic sites, does not preclude the presence of the other mutation in a different location. The presence of distinct mutations among samples of the same patient suggests that there might be different tumor clones in a primary tumor and as the disease progresses the mutational profile of the clones is modified.

Since only *KRAS* and *NRAS* mutations preclude patients from being treated with EGFR inhibitors, and in clinical practice most mutational status studies are performed in primary tumors [8], in our series, 20 of the 26 patients would have been classified as WT, and thereby candidates for anti-EGFR treatments. Of these 20 WT primary tumor patients, five had mutations in *KRAS* or *NRAS* in lymph nodes or liver metastases, which means that if only primary tumors had been analyzed when looking for mutations in *KRAS* and *NRAS*, five mutant, EGFR inhibitor-resistant cases would have been misdiagnosed. The presence of different tumor clones in a primary malignancy and at its metastatic sites, along with clonal evolution in sCRC might explain why some patients do not respond to EGFR inhibitors, and confirm the utility of evaluating *KRAS* and *NRAS* mutations not only in primary tumors, but also at all metastatic sites.

In summary, in this study we confirm the presence of different mutational profiles in primary tumors, lymph node metastases and liver metastases. We believe that the presence of acquired mutations in genes involved in the EGFR pathway at metastatic sites could explain why not all patients respond to EGFR. These results highlight the need to analyze the mutations in all available tumoral samples of the patient before deciding upon anti-EGFR treatment. Larger studies assessing the therapeutic impact of the mutation portrait detected in the different tumor samples of the same patient are needed.

## MATERIALS AND METHODS

### Patients and samples

Tissue specimens from 26 sporadic colorectal carcinomas, 16 paired lymph node metastases and 34 paired liver metastases (n=76 samples) were obtained from 26 patients (19 males and 7 females) before any systemic treatment or local radiotherapy was given. The median age was 67 years (range, 48-79 years). All patients had undergone surgical resection of tumor tissues (primary tumors, metastatic lymph nodes and liver metastases) at the Department of Surgery of the University Hospital of Salamanca (Salamanca, Spain) between 2000 and 2013, and they were recruited into the study after they had given their informed consent to participate. The study underwent institutional review and was approved by the Local Ethics Committee of the University Hospital of Salamanca (Salamanca, Spain).

Salient clinical and laboratory data of the 26 patients studied are fully described in Table 1 and summarized in Supplementary Table 1. All tumors were diagnosed and classified according to the WHO criteria [36] and staged according to the TNM Classification of Malignant Tumours (6th edition) [37]. By tumor grade, there were 16, 8 and 2 cases of well, moderately and poorly differentiated carcinomas. The histopathological grade of all tumors was confirmed in a second independent evaluation by an experienced pathologist.

Eleven primary tumors were localized in the rectum and the other 15 were in the right (caecum, ascending or transverse) or left (descending or sigmoid) colon. The median size of the primary tumors was 5.3 cm (range, 2.5-9.0 cm) with the following distribution by TNM stage: T2N0M0, 1 tumor; T3N0M0, 1; T3N0M1, 4; T3N1M0, 2; T3N1M1, 8; T3N2M0, 1; T3N2M1, 3; T4N0M0, 1; T4N0M1, 2; T4N1M1, 1; and; T4N2M1, 2 tumors. Paired liver metastases were identified at the time of colorectal surgery or during the first year after initial diagnosis (n=25); the mean size of the largest liver metastases was 4.0 cm (range, 0.5-10.0 cm).

Once the histopathological diagnosis had been established, sections from paraffin-embedded tissue samples were cut from three representative areas of the tumor tissue with >70% tumor cell infiltration as established by hematoxylin-eosin staining, after excluding stroma-enriched tumor areas. In order to enrich the tumor cells, the neighboring areas of those containing ≥70% tumor cells were then microdissected from the paraffin-embedded tumor tissue samples by an experienced pathologist. DNA was extracted and isolated using a Maxwell® 16 System for Genomic DNA Extraction (Promega, Mannheim, Germany) and quantified using a Qubit dsDNA BR assay (Invitrogen, Life Technologies, CA, USA).

### Mutational analysis using low-density microarray technology

After the histopathological diagnosis, primary tumor, lymph node metastasis and paired liver metastasis of each patient were tested for mutations in the *KRAS*, *NRAS*, *BRAF* and *PI3K* genes using a multiplex allele-specific PCR-based assay, which assesses 44 mutations in *KRAS* codons 12, 13, 59, 61, 117 and 146 (G12A, G12C, G12D, G12R, G12S, G12V, G13D, A59E, A59G, A59T, Q61K (C>A), Q61K (C>AA), Q61L, Q61R, Q61H(A>T), Q61H(A>C), K117N(A>C), K117(A>T), A146P, A146V, and A146T), *NRAS* codons 12, 13, 59, 61, 117 and 146 (G12D, G12C, G12S, G12A, G12V, G13D, G13R, G13V, A59T, Q61K, Q61R, Q61L, Q61H(A>C), Q61H(A>T), K117N(G>C), K117N(G>T), A146T and A146V)) and *BRAF* codon 600 (V600E(T>A), V600E(G>AA), V600D, V600K and V600R). A total of 76 assays (26 primary tumors, 16 lymph node metastases and 34 liver metastases) were performed using two kits based on polymerase chain reaction amplification and array hybridization with different probes, following the manufacturer's instructions (CLART^®^ CMA·KRAS·BRAF·PIK3CA and CLART CMA·NRAS·iKRAS kits; Genomica SAU Technology, Madrid, Spain). Estimated sensitivity was 1% as established by the manufacturer.

### Patient and public involvement

This study analysed cancer tissues from de-linked database. Therefore, we did not inform or disseminate to patients the research question, the outcome measures and the results. Patients were not involved in the study, including in the design, recruitment and conduct of the study. No patient adviser was included in the contributorship statement.

### Statistical methods

Means, standard deviations (SDs) and ranges of continuous variables, and the frequencies and percentages of dichotomous variables were calculated using IBM SPSS for Windows version 20.0 (IBM Corp., Armonk, NY, USA).

## SUPPLEMENTARY MATERIALS FIGURES AND TABLES


