# Supplementary material for: Spatio-temporal tumor heterogeneity in metastatic CRC tumors: a mutational-based approach
Source: Oncotarget. 2018 Sep 28;9(76):34279–88. doi: 10.18632/oncotarget.26081 (PMC6188146; doi:10.18632/oncotarget.26081)
Supplement: Supplementary file 1 [file oncotarget-09-34279-s001.pdf]

Spatio-temporal tumor heterogeneity in metastatic CRC tumors: a mutational-based approach

SUPPLEMENTARY MATERIALS

Supplementary Table 1: Clinical and biological characteristics of sporadic colorectal cancer (sCRC) patients (n=26)

Clinical Features	Total (n=26)
**Age (years)***	67 (48-79)
**Gender**	
Female	7 (27%)
Male	19 (73%)
**Site of primary tumor**	
Right colon	4 (16%)
Left colon	11 (42%)
Rectum	11 (42%)
**Histological type**	
Adenocarcinoma	25 (96%)
Adenocarcinoma with signet ring cells	1 (4%)
**Grade of differentiation**	
Well-differentiated	16 (61%)
Moderately differentiated	8 (31%)
Poorly differentiated	2 (8%)
**Histopathological tumor classification**	
pT2	1 (4%)
pT3	19 (73%)
pT4	6 (23%)
**Histopathological lymph node status**	
pN0	9 (35%)
pN1	11 (42%)
pN2	6 (23%)
**Metastatic status**	
M0	6 (23%)
M1	20 (77%)
**Stage at diagnosis**	
Stage I	1 (4%)
Stage IIA	1 (4%)
Stage IIB	1 (4%)
Stage IIIB	3 (11%)
Stage IV	20 (77%)
**Tumor size (cm)***	5.3 (2.5-9)
**CEA serum levels***	204 (0.8-1484)
≤5 ng/ml	4 (15%)
>5 ng/ml	22 (85%)

*Results expressed as median (range), or number of cases (percentage).
CEA: carcinoembryogenic antigen.
